# Incorporation of subject-level covariates in quantile normalization of miRNA data

**DOI:** 10.1186/s12864-015-2199-4

**Published:** 2015-12-09

**Authors:** Anvar Suyundikov, John R. Stevens, Christopher Corcoran, Jennifer Herrick, Roger K. Wolff, Martha L. Slattery

**Affiliations:** Department of Mathematics and Statistics, Utah State University, 3900 Old Main Hill, Logan, 84322-3900 UT U.S.A; BioStat Solutions, Inc., 5280 Corporate Drive, Suite C200, Frederick, 21703 MD U.S.A; Division of Epidemiology, Department of Internal Medicine, University of Utah School of Medicine, 383 Colorow Road, Salt Lake City, 84108 UT U.S.A

**Keywords:** Normalization, Differential expression, miRNA

## Abstract

**Background:**

Most currently-used normalization methods for miRNA array data are based on methods developed for mRNA arrays despite fundamental differences between the data characteristics. The application of conventional quantile normalization can mask important expression differences by ignoring demographic and environmental factors. We present a generalization of the conventional quantile normalization method, making use of available subject-level covariates in a colorectal cancer study.

**Results:**

In simulation, our weighted quantile normalization method is shown to increase statistical power by as much as 10 % when relevant subject-level covariates are available. In application to the colorectal cancer study, this increase in power is also observed, and previously-reported dysregulated miRNAs are rediscovered.

**Conclusions:**

When any subject-level covariates are available, the weighted quantile normalization method should be used over the conventional quantile normalization method.

**Electronic supplementary material:**

The online version of this article (doi:10.1186/s12864-015-2199-4) contains supplementary material, which is available to authorized users.

## Background

A critical data analysis step in detecting differentially expressed microRNA (miRNA) features is normalization. The normalization procedure reduces technical variation and maintains true biological changes between arrays. Various normalization techniques exist, but until recently, all were developed for messenger RNA (mRNA) arrays. The miRNA data are very different from mRNA data due to the small total number of miRNAs (a few hundred versus 10,000 to 50,000 genes in mRNA data), and the majority of miRNAs are either not expressed or are expressed at very low levels [1]. Therefore, normalization methods used for mRNA expression arrays may not be appropriate for miRNA arrays. More information about the biology of miRNAs and their role in cancer development is given in Suyundikov [2] and Suyundikov et al. [3], which also present the colorectal cancer (CRC) study motivating this paper. Briefly, the study data used here has miRNA data from paired tumor and normal samples in over 400 subjects, with over 2,000 miRNAs. (These are the first available subjects from about 2,000 subjects in the overall study.) The study was approved by the Institution Review Board for Human Subjects at the University of Utah (IRB_00002335 and IRB_00055877). All participants provided informed written consent prior to participating in the study.

The importance of finding an appropriate normalization method for miRNA data in our colorectal cancer (CRC) study [3] motivated us to develop a normalization method that accounts for the characteristics of data, removes any artificial variations, and keeps the crucial biological information. CRC is the third most common type of cancer and the second leading cause of cancer death in the United States [4]. Most colorectal cancers are due to demographic, lifestyle, and health-related factors, with only a small number of cases due to underlying genetic disorders [5, 6]. Cunningham et al. [7] and Watson and Collins [8] listed older age, male gender, high intake of fat, alcohol or red meat, obesity, smoking, and a lack of physical exercise as risk factors of CRC. Taken together with known and hypothesized associations of miRNA with CRC, this suggests that the expression levels of miRNAs in tissues from risk group (for example, older and smoker) patients are more likely to be differentially expressed than the expression levels from non-risk group (younger and non-smoker) patients.

In our CRC study, we have collected extensive information about demographic and lifestyle variables of CRC patients along with the miRNA features from normal and tumor samples. These data may be helpful to consider not only the artificial intra- and inter-array differences, but also the differences caused by the demographic and lifestyle characteristics of patients, and to maintain only biological differences during the normalization procedure.

In this paper, we incorporate subject-level covariates (specifically the demographic and lifestyle variables) in miRNA normalization, which has not been done before. We modify the quantile normalization method from Bolstad et al. [9] that is commonly used in miRNA data analysis and was found as an efficient method to remove the artificial differences across arrays [10–14]. The quantile normalization equalizes the distributions of expression intensities across samples while ignoring any differences of characteristics of samples. In the quantile normalization method, each subject’s normalized distribution of expression values depends on all other subjects’ distributions equally. Such normalization of miRNA expressions while ignoring the characteristics of data results in loss of important biological information. In our modified normalization method, we assume that the normalized distribution of miRNAs from one subject should depend on the weighted distribution of miRNAs from other subjects. The weights of subjects are determined from the distance matrix generated from various distance metrics of multiple covariates. The elements of the generated distance matrix represent the pairwise distances between two subjects based on demographic and lifestyle variables. The distances (weights) among subjects are considered in the quantile normalization of miRNA.

This paper is arranged in the following manner: first, we provide an overview of normalization methods developed for miRNA data and explain our modified normalization method in detail. Then we show the application of normalization techniques using simulation and real data sets. Finally, we conclude with a discussion of the important findings presented in this paper.

## Methods

### Normalization methods for miRNA data

Normalization (along with background correction and summarization [14]) is one of the important steps of preprocessing of miRNA data. The procedure removes the systematic differences between arrays that do not represent true biological variation. Meyer et al. [15] state that “normalization, often an underestimated aspect of data processing, can minimize systematic technical or experimental variation and thus has significant impact on the detection of differentially expressed miRNAs.” Bolstad et al. [9] highlight that the need for normalization arises naturally when multiple arrays are involved in experiments. There are two types of variations that can be expected: the first variation is an “interesting” variation, which represents biological differences between the expression levels of genes (or miRNAs) from tumor and normal tissues (for example, as in our CRC study), and the other is an “obscuring” technical variation, which is not interesting for the researchers and needs to be removed by a normalization procedure. Currently used normalization methods in miRNA data analysis were primarily developed for mRNA arrays, which have an exceedingly high density of probes with 10,000–50,000 genes. In comparison, miRNAs are lower density arrays with a few hundred to a couple of thousand genes. Wang and Xi [1] mentioned that the majority of miRNAs are either not expressed or are expressed at very low levels. Therefore, researchers have generally concluded that off-the-shelf normalization methods for mRNA arrays may not be appropriate for miRNA arrays [11, 13, 15].

Several studies have compared the performance of normalization methods for mRNA data to see how these methods can reduce the experimentally induced variation and maintain true biological changes between arrays in miRNA data analysis. Rao et al. [11] applied commonly used normalization methods, including cyclic loess, quantile, median or mean, and no normalization techniques to normalize miRNA expression arrays. Their analyses show that the quantile normalization method works better than other normalization techniques in removing differences across arrays in miRNA expression data. Pradervand et al. [13] also examined the impacts of mRNA array normalization procedures based on scaling, quantile, and variance stabilizing normalization (VSN) on miRNA data. They found that the quantile normalization was the most robust procedure and performed at least as well as the developed normalization method based on the set of invariants (invariants-based) among the mRNA normalization techniques that they considered (including quantile, invariants-based, scaling, VSN, and no normalization methods) over all experimental conditions tested. All normalization methods performed better than no normalization. For this reason, we chose not to perform the no normalization technique along with other normalization methods in our analysis. Over the last decade, the quantile normalization method has been commonly used in miRNA data analysis compared to the other normalization techniques developed for mRNA data [10, 12, 14]. We explain the algorithm of quantile normalization in the “Conventional quantile normalization” section.

A number of modifications to normalization methods for mRNA data have been performed to adapt to the characteristics of miRNA data. Though each modified normalization method has been shown to perform well based on the characteristics of tested miRNA data [16–20], a universal normalization method for miRNA data has not been developed yet. Meyer et al. [15] strongly suggest selecting the optimal normalization method based on the characteristics of the data set, and then examining the normalized data carefully in specific biological contexts. The choice of normalization method is one of the primary factors that affects the inference of differential expression [18].

### Quantile normalization: conventional and modified to incorporate covariates

#### Conventional quantile normalization

Bolstad et al. [9] were among the first to apply quantile normalization to microarray data analysis. They compared its performance with the cyclic loess and contrast based normalization methods that had already been successfully used in mRNA data analysis. The purpose of the quantile normalization is to force the distribution of probe intensities for each array in a set of arrays to have the same or at least similar distribution. Bolstad et al. [9] were motivated by the idea that a quantile-quantile plot demonstrates the same distribution for two data vectors if the plot is a straight diagonal line, and not the same distribution if the plot is other than a diagonal line. They extended this concept to *N* dimensions of data vectors so that all data vectors have the same distribution. The quantiles of *N* data vectors (here, the intensities of *N* arrays) are plotted in such a way that (after normalization) the plot gives a straight line along the line given by the unit vector $\left (\frac {1}{\sqrt {N}}, \ldots, \frac {1}{\sqrt {N}}\right)$. To achieve this normalization, one can make the distribution of a set of data vectors the same if one projects the points of the *N* dimensional quantile onto the diagonal unit vector. More details about the projection of the quantiles onto the diagonal are provided in Bolstad et al. [9]. The numerical dependence induced by this normalization method is relatively minimal [21].

Bolstad et al. [9] provided the following algorithm to perform a quantile normalization: arrange the logarithmic transformed microarray data into a *G*×*N* matrix ${\underset {\sim }{X}}$, where *G* and *N* are total numbers of genes and arrays, respectively. Sort each column of ${\underset {\sim }{X}}$ to give ${\underset {\sim }{X}}_{\textit {sort}}$. Then take the means across the rows of ${\underset {\sim }{X}}_{\textit {sort}}$ and assign this mean to each element in the row to get ${\underset {\sim }{X}}_{\textit {sort}}^{\prime }$. At the end, obtain the normalized version ${\underset {\sim }{X}}_{\textit {norm}}$ of ${\underset {\sim }{X}}$ by rearranging each column of ${\underset {\sim }{X}}_{\textit {sort}}^{\prime }$ to have the same ordering as in the original ${\underset {\sim }{X}}$.

Another algorithm to carry out the quantile normalization was introduced by Amaratunga and Cabrera [22]. They described the algorithm as follows: “calculate the percentiles (*Q*_*i*0_,…,*Q*_*i*100_) of the *i*th array and the percentiles (*Q*_*M*0_,…,*Q*_*M*100_) of the median mock array. For any value *X*_*gi*_, find the interval, [*Q*_*ih*_,*Q*_*i*(*h*+1)_], to which it belongs and obtain its normalized value, $X_{\textit {gi}}^{\prime }$, by linearly interpolating between the pair points (*Q*_*Mh*_,*Q*_*ih*_) and (*Q*_*M*(*h*+1)_,*Q*_*i*(*h*+1)_)” [22]. In this algorithm, *X*_*gi*_ means the logarithmic transformed spot intensity measurement for gene *g* on array *i*. They define the median mock array as the array fashioned out of the medians of the arrays being normalized.

While the algorithm from Bolstad et al. [9] is more widely applied in practice than the algorithm of Amaratunga and Cabrera [22], no study has yet been published that evaluates their relative performance. In our analysis, we used the normalize.quantiles function from the R package *preprocessCore* [23] that is based upon the concept of the quantile normalization from Bolstad et al. [9]. We further refer to the quantile normalization based on the algorithm from Bolstad et al. [9] as the conventional quantile normalization.

#### Weighted quantile normalization

The conventional quantile normalization does not account for additional characteristics of samples when it normalizes the miRNA arrays. In this respect, we propose a novel approach that removes the non-biological differences across samples while incorporating the demographic and lifestyle characteristics of sample-subjects in normalization. Here, we assume that the normalized distribution of miRNA expression levels from one sample should depend on the weighted distribution from other samples. The weights of subjects are determined from a distance matrix, which is aggregated [2] from various normalized distance matrices (values are between 0 and 1 [24]; based on Euclidean, Manhattan, Minkowski, and other methods) of multiple covariates. The elements of the aggregated distance matrix (${\underset {\sim }{D}}$) represent the pairwise distances between two subjects based on relevant demographic and lifestyle variables.

The algorithm of our proposed method is based on the quantile normalization algorithm from Bolstad et al. [9], but accounts for the weighted distance metrics of demographic and lifestyle variables. The algorithm is as follows:

1. Obtain the logarithmic (log2-based) transformed miRNA data as a *G*×*N* matrix ${\underset {\sim }{X}}$, where *G* is the total number of miRNAs and *N* is the total number of subjects. The log-transformation of miRNA data is performed to reduce the effect of outliers on the calculation of miRNA expression levels.
2. Sort each column-subject of ${\underset {\sim }{X}}$ to give ${\underset {\sim }{{X}_{\textit {sort}}}}$.
3. Obtain an aggregated distance matrix as a *N*×*N* matrix ${\underset {\sim }{D}}$.
4. Obtain the between-subject weight matrix as a *N*×*N* matrix ${\underset {\sim }{W}}$, with elements *w*_*ij*_. We calculate the weight of subject *i* in the normalized expression distribution of subject *j* as in (1):
  (1) $$ w_{ij} = 1 - \frac{d_{ij}}{max\{d_{i1},\dots,d_{iN}\}}  $$
  where *d*_*ij*_ (an element of ${\underset {\sim }{D}}$) is the distance between subjects *i* and *j* with *i,j*=1,…,*N*. If *i*=*j*, then *w*_*ij*_=1. Note that the matrix ${\underset {\sim }{W}}$ is not symmetric and the elements of row *i* correspond to the weights of the *N* subjects in the normalized expression distribution of subject *i*.
5. Normalize the between-subject weight matrix ${\underset {\sim }{W}}$ by the sum of the weights of row-subjects and obtain a *N*×*N* matrix ${\underset {\sim }{W}}^{*}$. For example, the weighted means of weights of subjects in the normalized expression distribution of subject *i* can be found as in (2):
  (2) $$ w_{ij}^{*} = \frac{w_{ij}}{{\sum_{j\prime=1}^{N} w_{\textit{ij}\prime}}} \; \quad \text{s.t.} \; \quad \sum_{j=1}^{N} w_{ij}^{*} = 1.  $$
6. Calculate the weighted means across the rows of ${\underset {\sim }{{X}_{\textit {sort}}}}$ and assign this weighted mean to each element in the row to get a *G*×*N* matrix ${\underset {\sim }{{X}_{\textit {sort}}^{\prime }}}$.
  The weighted means of ${\underset {\sim }{X_{\textit {sort}}}}$ can be computed as in (3):
  (3) $$ {\underset{\sim}{{X}_{sort}^{\prime}}} = {\underset{\sim}{{X}_{sort}}} \left[ {\underset{\sim}{W}}^{*}\right]^{T}.  $$
7. Obtain the normalized version ${\underset {\sim }{X_{\textit {norm}}}}$ of ${\underset {\sim }{X}}$ by rearranging each column of ${\underset {\sim }{{X}_{\textit {sort}}^{\prime }}}$ to have the same ordering as in the original ${\underset {\sim }{X}}$.

The above mentioned algorithm generalizes the quantile algorithm of Bolstad et al. [9], in which all $w_{\textit {ij}}^{*}=\frac {1}{N}$. While the conventional quantile normalization method equally weights all subjects, this weighted quantile normalization method instead weights subjects according to their similarity to each other. That is, the weighted-normalized expression values for a given subject are affected more by the expression values of similar subjects than by those of unsimilar subjects. This weighted quantile normalization algorithm is implemented (with a demonstration using simulated data) in code written for the R language [25], and is provided as Additional file 1 (see “Additional files” section).

In contrast to the normalization methods for miRNA data where disjoint clusters of miRNAs were considered (Mestdagh et al. [26], Bargaje et al. [27]), this normalization technique will consider both clustered and overlapped subjects by accounting for each subject’s weight in the average. The clustered subjects will have heavier weights in the average than the weights of subjects who are not clustered. This normalization technique will contribute to reduction of intra- and inter-array technical variability while maintaining biological differences. We subsequently refer to this proposed method as the weighted quantile normalization.

## Results and discussion

In this section, we demonstrate the performance of the weighted quantile normalization method over the conventional quantile normalization method using different simulation scenarios.

### Motivating example

**Fig. 1:**
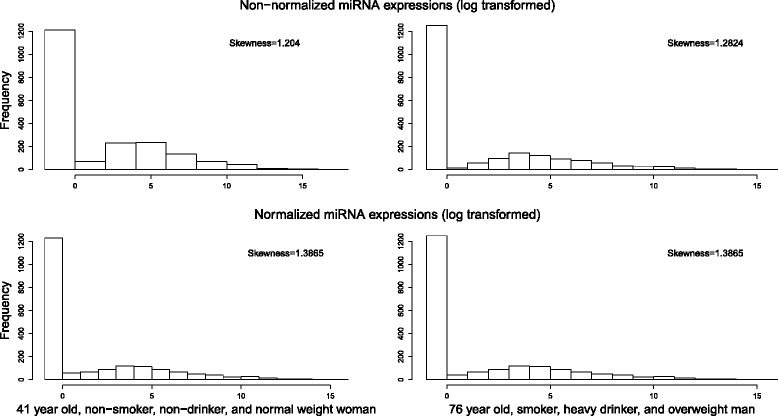
Non-normalized and quantile normalized miRNA expressions of tumor samples from non-risk and risk group subjects

Figure 1 illustrates the potential danger of normalizing without regard for relevant demographic or environmental factors. The miRNA distributions for two CRC subjects are displayed. For each individual (one from the “non-risk” and one from the “at-risk” sample), both the non-normalized and conventionally quantile-normalized distributions are plotted. The first (non-risk) subject is a 41-year-old, non-smoking, non-drinking, normal-weight woman, while the second (at-risk) is a 76-year-old, current-smoking, heavy drinking, overweight man. The non-normalized miRNA expression histograms, at the top of Fig. 1, show clear disparities between the two subjects. The distribution of the risk-group patient is clearly more variable and right-skewed in comparison to the miRNA distribution of the non-risk patient. (Skewness is quantified here in terms of the Fisher-Pearson coefficient of skewness, and reported in Fig. 1.) However, after performing the quantile normalization of miRNA expressions, both subjects have almost indistinguishable distributions. This suggests that their similarity is merely an artifact of the normalization, and that important differences in gene expression could be masked by ignoring demographic and environmental factors. Our repeated observation of such examples has motivated our development of the novel weighted quantile normalization method (see the “Weighted quantile normalization” section) that properly removes any technical variations, while preserving important biological information with regard to expression differences, and further allowing us to account for additional covariates.

We evaluate the performance of our proposed weighted quantile normalization method using simulated data in sections “Simulation data sets” through “Normalization accounting for unrelated covariates”. We return to the motivating example in the “Application to motivating example (real CRC data)” section.

### Simulation data sets

The normalization analyses were carried out on bimodally distributed paired data matrices of *G*=2000 miRNA expression features (rows) for each of the normal and tumor samples with sample sizes of *N*=200 and 400 subjects (columns). We simulated expression levels of miRNAs for normal and tumor samples by controlling true differentially expressed miRNAs of tumor samples across all simulations. The simulated bimodal miRNA data sets were generated by the mixture of two normal distributions, and reflect the two modes (for non-expressed and expressed features) generally seen in miRNA data. Particularly, all miRNA features of normal samples and only non-differentially expressed miRNA features of tumor samples were simulated based on *μ*=0.75 and *σ*=0.025 for the first distribution and *μ*=4.0 and *σ*=0.5 for the second distribution, while the differentially expressed miRNA features of tumor samples, which consisted of 20 % of all miRNA features of tumor samples, were simulated based on *μ*=0.75 and *σ*=0.025 for the first distribution and *μ*=3.25 or *μ*=4.75 and *σ*=0.5 for the second distribution. These parameters were chosen based on observed characteristics of our CRC study data. We performed 20 simulations for each sample size.

Moreover, we simulated demographic and lifestyle variables of subjects in such a way that they could reflect the characteristics of our CRC study and also carry some useful information for the normalization procedure. In our analysis, we simulated all available 19 noncollinear demographic and lifestyle variables (as listed in Tables 1 and 2) from the CRC study. Briefly, the covariates’ values were simulated to be associated with the expression values of several randomly-selected miRNAs that were controlled as truly differentially expressed between tumor and normal; for details, see Suyundikov [2] and Suyundikov et al. [3].

**Table 1:** Summaries of continuous covariates in real CRC data

Covariate	Mean	SD
Age at diagnosis or selection (years)	64.1	9.8
Average num. cigarettes per day	12.5	14.7
Calories (kcal)	2504.7	1199.3
BMI	27.6	5.4
lutein + zeaxantin (mcg)	3119.3	2542.3
Vitamin D (mcg)	6.7	5.0
Lycopene (mcg)	8850.5	8195.1

**Table 2:** Summaries of binary or discrete covariates in real CRC data

Covariate	Summary
Gender	54 % male, 46 % female
Recent aspirin/NSAID use	64 % no, 36 % yes
Recent smoker	83 % no, 17 % yes
(among women) menopause	12 % pre, 88 % post
(among post-menopausal women)	
taking HRT within 2 years	30 % yes, 70 % no
Data collection center	79 % Kaiser, 21 % Utah
Race	81.6 % White, 8.5 % Hispanic,
	7.6 % Black, 2.1 % other
Smoking status	13 % current, 45 % former,
	42 % never
Long-term alcohol consumption	38 % none, 35 % moderate,
	27 % high
SEER summary stage	1 % in situ, 34 % localized, 52 %
	regional, 12 % distant, 1 % unknown
AJCC severity stage	1 % 0 (in situ), 26 % 1, 31 % 2, 30 % 3,
	12 % 4 (distant)
Colon or rectal cancer	76 % colon, 24 % rectal

We started the simulation analyses by generating miRNA expressions of normal and tumor samples and demographic and lifestyle variables based on the simulation parameters mentioned above. During simulation of miRNA expressions, we controlled arbitrarily 20 % of miRNAs from tumor samples as differentially expressed features. Euclidean distance was used for continuous covariates and Manhattan distance for discrete or binary covariates. The two Euclidean and Manhattan between-subject distance matrices were normalized by scaling between 0 and 1 [24] and aggregated into a single between-subject distance matrix by taking their weighted average [2].

As an aside, the application of the weighted quantile normalization method is not computationally burdensome; it took less than one minute to normalize the expressions of 2000 miRNAs from 400 subjects on a machine with CPU speed of 1.86 GHz and 2 GB RAM.

### Differential expression testing

We carried out the normalization methods mentioned in the “Methods” section. We conducted the differential expression analyses on tumor-normal differences (using a per-miRNA Wilcoxon signed rank test, or SRT) on the normalized data sets to check whether the weighted quantile normalization method has an equal statistical power in finding differentially expressed miRNA as the conventional quantile normalization. First, we obtained the Wilcoxon SRT statistic and *p*-value for each miRNA feature in each normalized data set and controlled the false discovery rate (FDR) at 0.05 within each simulation. Then, we calculated the true positive rate (TPR) and the false discovery rate (FDR) based on the miRNAs which were controlled as truly differentially expressed in the simulations. The TPR was defined and calculated as in Bolstad [28] and Stevens et al. [29], and the FDR was defined as in Benjamini and Hochberg [30].

**Fig. 2:**
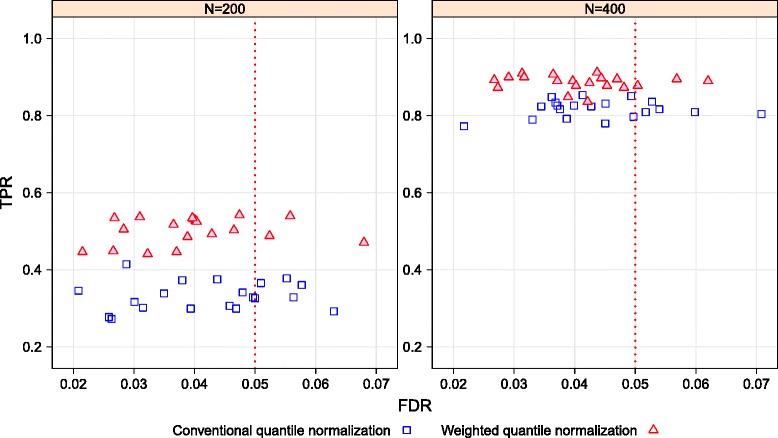
TPR and FDR for sample sizes of 200 and 400 for conventional and weighted quantile normalizations

Figure 2 shows the performance (including power and FDR control) of the Wilcoxon SRT on the data sets normalized by the conventional quantile and the weighted quantile methods for the numbers of subjects of 200 and 400. As shown in this scatter plot, the power (i.e., the TPR values) increases (as would be expected) for the conventional quantile (a blue open rectangular symbol) and the weighted quantile (a red solid triangular symbol) normalization methods with larger sample sizes. The weighted quantile normalization has clearly higher power than the conventional quantile method. For 400 subjects, which is more similar to the size of our CRC study, the differences of TPR values between the two methods are up to 10 %: the power for the weighted quantile normalization is in the range of 83.6–90.5 %, while the power for conventional one is in 77.2–85.3 %. That is, one can increase power as much as 10 % by using the weighted quantile normalization method rather than the conventional quantile method. Both normalization methods generally control the FDR near 0.05 for both sample sizes. Results were generally similar when Manhattan distance was used for all covariates (see Additional file 2), rather than Euclidean for continuous covariates and Manhattan for discrete (as in Fig. 2).

### Normalization accounting for unrelated covariates

**Fig. 3:**
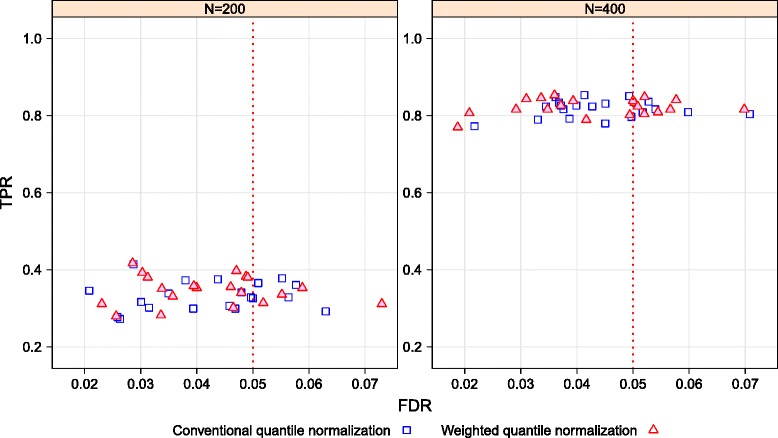
TPR and FDR for sample sizes of 200 and 400 for the quantile normalization and the weighted quantile normalization while accounting for unrelated covariates

The application of the weighted quantile normalization method only increases power (compared to conventional quantile normalization) when demographic and lifestyle variables (on which between-subject distance is based) are relevant to the “treatment” group comparison of interest. To demonstrate this, we again simulated the 19 covariates, but this time only as noise, without any reference to the miRNA data as in Suyundikov et al. [3]. Figure 3 shows the performance (the TPR versus the FDR) of the Wilcoxon SRT on the simulated data sets that are normalized by the conventional quantile and the weighted quantile methods while accounting for these unrelated (pure noise) covariates. The power and the FDR control are essentially the same (overlap in most simulations) for both normalization methods. Thus, the weighted quantile normalization performs at least as well as the conventional quantile normalization when demographic and lifestyle variables are not associated with the treatment group. Results were generally similar when Manhattan distance was used for all covariates (see Additional file 3), rather than Euclidean for continuous covariates and Manhattan for discrete (as in Fig. 3).

### Application to motivating example (real CRC data)

We used the Wilcoxon SRT to identify differentially expressed miRNAs in the paired tumor-normal miRNA data from our CRC study [3]. The miRNA data were normalized by the conventional quantile and by the weighted quantile methods while accounting for the demographic and lifestyle characteristics of CRC subjects. These data sets contain the first available 527 subjects with 2006 miRNA on each sample. In this analysis, we used all available 19 noncollinear demographic and lifestyle variables, as summarized in Tables 1 and 2.

**Fig. 4:**
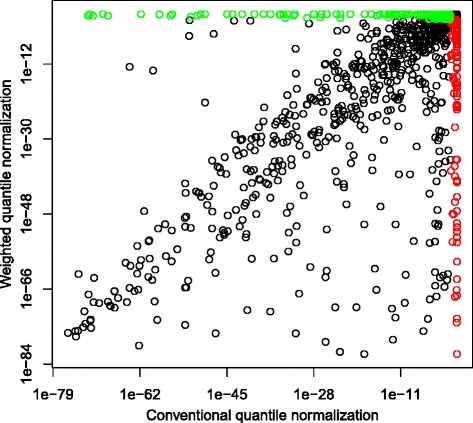
Scatter plot of adjusted *p*-values of the CRC miRNA data, normalized by the quantile and the weighted quantile normalization methods (in log-scale). The green and red circles represent the miRNAs that are found significant only in the horizontal and vertical analyses, respectively

Figure 4 shows a scatter plot of the FDR adjusted *p*-values in logarithmic scale. The green circles (there are 121) in the plot represent the miRNAs that are found significant from the quantile normalized data, but not found significant from the weighted quantile normalized data. The red circles (there are 119) show the miRNAs that are found significant only in the weighted quantile normalized data. There is no information about the truly differentially expressed miRNAs that could be helpful to evaluate the performances of both normalization methods. However, we can see from Fig. 4 that many miRNAs (in the lower right triangle of the plot) that are found significant in the quantile normalized data are found to be even more significant in the data set normalized by the weighted quantile method. The plot shows that the proposed weighted quantile normalization method has more power than the conventional quantile method.

By way of validation, our results (from the use of the weighted quantile normalization method) can be placed in the context of recent CRC miRNA studies by comparing the resulting list of candidate miRNAs with those published by Dong et al. [31] and Mazeh et al. [32]. Specifically, we focus on those miRNAs reported in Table 2 of [31] as being prognostic or predictive markers for CRC, and those miRNAs reported in Table 1 of [32] as dysregulated (by at least four studies) in colorectal tissue samples. A total of 41 miRNAs were thus considered.

Due to changes over time in miRNA naming schemes and platform capabilities, any direct comparison of results is inherently uncertain. For example, what was previously reported as miR-203 (up-regulated in tumor [32]) could appear in our CRC study as miR-203a (up-regulated in tumor), miR-203b-3p (no significant difference in tumor), or miR-203b-5p (no significant difference in tumor). Similarly, what was previously reported as miR-195 (down-regulated in tumor [32]) could appear in our CRC study as miR-195-3p (up-regulated in tumor) or miR-195-5p (down-regulated in tumor). With this in mind, and assuming that what previous studies reported for a general-named miRNA was actually what we found for more specific-named miRNA(s), we can report that of those 41 miRNAs previously reported [31, 32] as differentially expressed in tumor compared to normal colerectal tissue, we reach the same conclusion (for both statistical significance and direction of dysregulation) for 38 miRNAs. (This 93 % validation rate is summarized in Additional file 4.) Of the three remaining discrepancies, one (miR-1 down-regulated in tumor [32]) we found with marginal significance (FDR-adjusted *p*-value.08), and the other two involved possible naming scheme discrepancies. Specifically, miR-106a was previously reported as up-regulated in tumor [31, 32], and our CRC study found no significant difference for miR-106a-3p (FDR-adjusted *p*-value 1), and did not measure miR-106a-5p (which perhaps was the miRNA actually studied previously). Also, miR-30a-3p was previously reported as down-regulated in tumor [32]; while we found no significant difference in miR-30a-3p (FDR-adjusted *p*-value 1), we did find miR-30a-5p significantly down-regulated (FDR-adjusted *p*-value < 0.0001). In short, the weighted quantile normalization method allowed the rediscovery of nearly all of the dysregulated miRNAs previously reported by [31] and [32].

In the 38 dysregulated miRNAs rediscovered here, the weighted quantile normalization results tended to have lower *p*-values than those from the conventional quantile normalization (Additional file 4), illustrating the greater power alluded to in Fig. 4.

## Conclusion

In this paper, we modified the quantile normalization method from Bolstad et al. [9] to reduce not only the artificial variations across samples, but also the variations caused by the characteristics of data, as well as to maintain true biological differences across samples. The proposed normalization method incorporates demographic and lifestyle variables by considering the distances (weights) among subjects based on their characteristics, and considers these weights in equating the quantiles of distributions of miRNA expressions. Accounting for such subject-level covariates in the normalization step helps avoid the masking of important expression differences, which can otherwise occur with conventional quantile normalization (Fig. 1).

We performed differential expression tests on simulated bimodally distributed miRNA expressions and demographic and lifestyle variables that reflect the characteristics of our CRC study. We applied the Wilcoxon SRT to identify differentially expressed miRNAs from the normalized simulated miRNA data. The scatter plots of TPR versus FDR help to evaluate the impact of normalization techniques on the results of the differential expression test (Fig. 2). The simulation study provides strong evidence that our weighted quantile approach yields up to a 10 % gain in power in comparison to the conventional normalization method. Both methods generally control the FDR near 0.05 for both sample sizes, and the weighted quantile normalization method is computationally convenient.

When irrelevant subject-level covariates are used in weighted quantile normalization, power and FDR control are essentially the same as when conventional quantile normalization is used (Fig. 3). This suggests that whenever subject-level covariates are available, weighted quantile normalization should be used because it is at least as good as conventional quantile normalization (in terms of power and FDR control), but substantially better in the presence of relevant demographic or environmental factors.

For the differential expression tests on the paired tumor-normal miRNA data from our CRC study, the Wilcoxon SRT found many miRNAs which were called significant in the conventional quantile normalized data, even more significant in the data set normalized by the weighted quantile method (Fig. 4).

## Additional files

Additional file 1
**Provides an implementation of the weighted quantile normalization algorithm (as describe in the “** **Weighted quantile normalization** **” section) written for the R language [** 25 **], with a demonstration using simulated data.** (R 2 kb)

Additional file 2
**Reproduces Fig.** 2 **, but with Manhattan distance used for all covariates (rather than Euclidean for continuous covariates and Manhattan for discrete, as in Fig.** 2 **).** (PDF 6 kb)

Additional file 3
**Reproduces Fig.** **3** **, but with Manhattan distance used for all covariates (rather than Euclidean for continuous covariates and Manhattan for discrete, as in Fig.** **3** **).** (PDF 5 kb)

Additional file 4
**Summarizes the reproduction of significance results from previous CRC miRNA literature [** 31 **,** 32 **] as discussed in the “** **Application to motivating example (real CRC data)** **” section, using the weighted quantile normalization method.** (XLSX 16 kb)
