# Supplementary figures and images for: Incorporation of subject-level covariates in quantile normalization of miRNA data
Source: BMC Genomics. 2015 Dec 9;16:1045. doi: 10.1186/s12864-015-2199-4 (PMC4675058; doi:10.1186/s12864-015-2199-4)

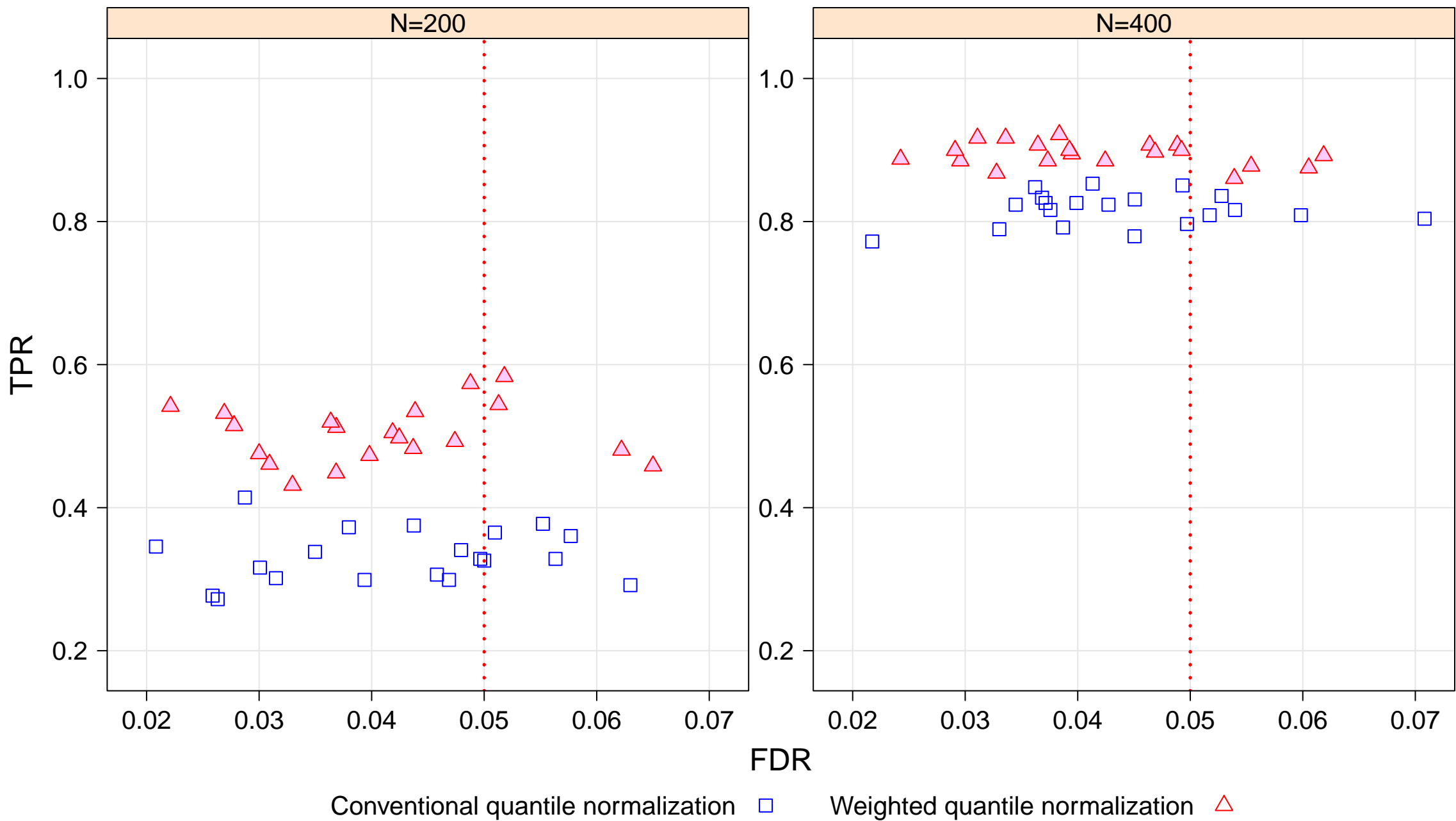

Supplement: Additional file 2 — Reproduces Fig. 2 , but with Manhattan distance used for all covariates (rather than Euclidean for continuous covariates and Manhattan for discrete, as in Fig. 2 ). (PDF 6 kb) [file 12864_2015_2199_MOESM2_ESM.pdf]

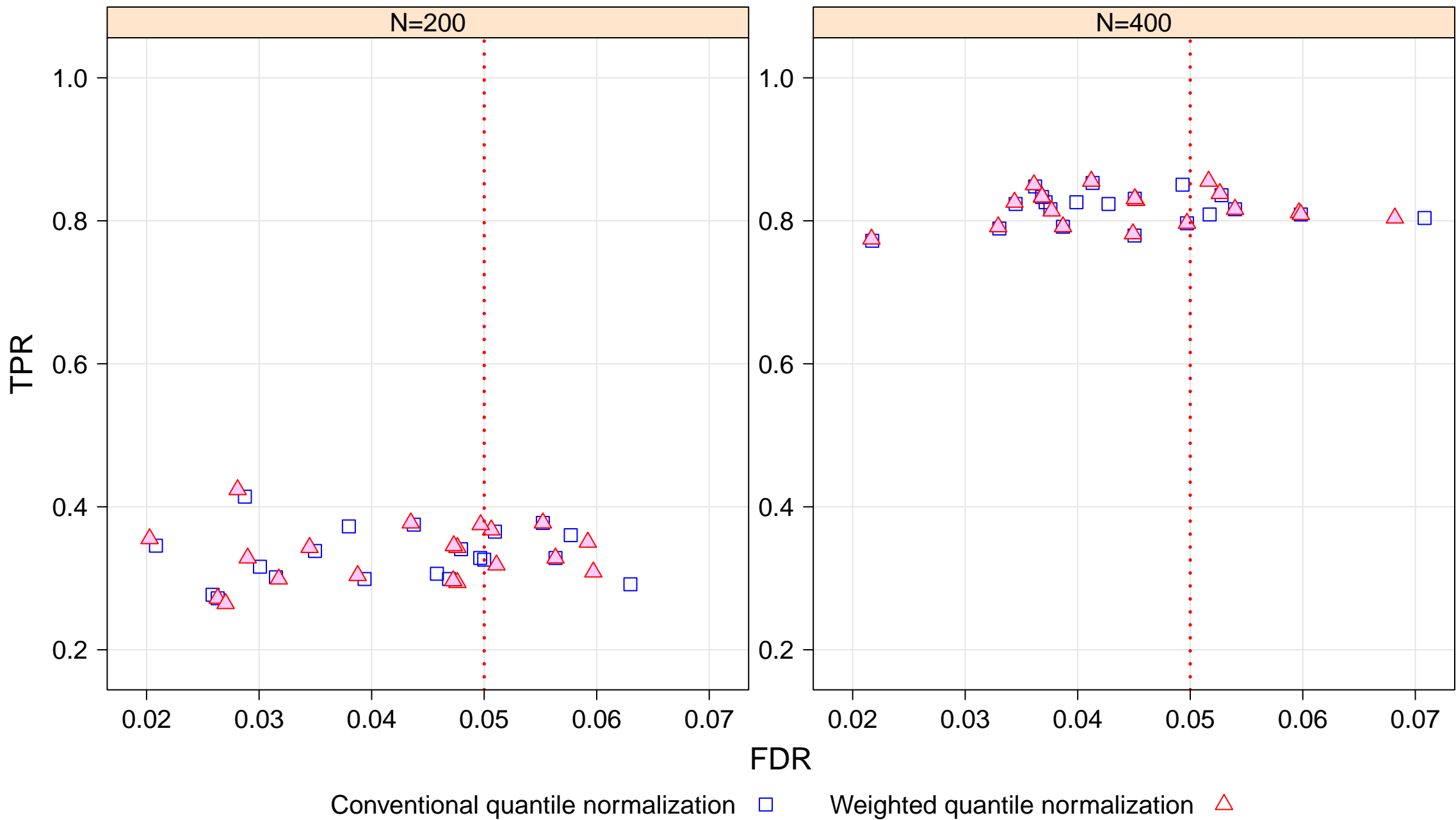

Supplement: Additional file 3 — Reproduces Fig. 3 , but with Manhattan distance used for all covariates (rather than Euclidean for continuous covariates and Manhattan for discrete, as in Fig. 3 ). (PDF 5 kb) [file 12864_2015_2199_MOESM3_ESM.pdf]
